# Fast-track anesthesia and outcomes in hepatopancreatic cancer surgery: a retrospective analysis

**DOI:** 10.1186/s44158-024-00152-8

**Published:** 2024-02-23

**Authors:** Sebastiano Mercadante, Fabrizio David, Lucio Mandalà, Patrizia Villari, Pietro Mezzatesta, Alessandra Casuccio

**Affiliations:** 1 Anesthesia & Intensive Care Unit, La Maddalena Cancer Center, Via san, Lorenzo 312, 90145 Palermo, Italy; 2 Department of Surgery, La Maddalena Cancer Center, Palermo, Italy; 3 Department of Experimental Biomedicine and Clinical Neuroscience, University of Palermo, Palermo, Italy

**Keywords:** Anesthesia, Fast-track, Epidural analgesia, Liver surgery, Pancreatic surgery, Cancer

## Abstract

**Aim:**

To assess the feasibility of a fast-track anesthesia protocol for hepatopancreatobiliary cancer surgery.

**Methods:**

Retrospective analysis of consecutive sample of patients who underwent hepatopancreatic surgery for cancer for a period of 12 months in a high volume cancer center. Blended anesthesia was performed for most patients who were then observed in a recovery room area until achieving a safety score.

**Results:**

Data of 163 patients were examined. Fifty-six and 107 patients underwent surgery for pancreatic cancer and liver surgery for primary tumor or metastases, respectively. Most patients were ASA 3. The mean durations of anesthesia and surgery were 322 min (SD 320) and 296 min (SD 133), respectively. Extubation was performed in the operating room in 125 patients. Post-operatory invasive ventilation was maintained in the recovery room in fifteen patients for a mean duration of 72.7 min (SD148.2). Only one patient was admitted to intensive care for 15 h. NIV was performed in three patients for a mean duration of 73.3 min (SD 15.3). The mean recovery room staying was 79 min (SD 80). The mean hospital postoperative stay was a mean of 8.1 days (SD 5.7). No complications were found in 144 patients. Globally, mortality rate was 3%.

**Conclusion:**

A program of fast-track anesthesia with a short stay in recovery room allowed to achieve a good outcome, limiting the costs of intensive care admission.

## Introduction

Multimodal perioperative management may hasten recovery and decrease morbidity and hospital stay following major surgical procedures [1–3]. Preoperative patient information, specific modalities to provide intraoperative and postoperative analgesia, and careful monitoring in the recovery room may result in a fast discharge to the surgical unit, without requiring intensive care unit admission. Patients undergoing pancreatic and liver surgery for cancer are considered as high-risk subjects to be cared for in specialistic centers. There are few published data for enhanced recovery after liver or pancreatic surgery. Enhanced recovery after surgery (ERAS) pathways target specific areas within perioperative patient care in a multidisciplinary and evidence-based manner and have expanded to most surgical subspecialties, including hepatopancreatobiliary surgery [4, 5]. A fast-track program, according to principal ERAS recommendations, has been optimized in our department to keep the costs down while providing the optimal outcome for cancer patients with liver or pancreatic cancer. The aim of this retrospective study was to analyze the outcomes of a protocol of fast-track anesthesia for major abdominal cancer procedures including pancreatic and liver surgery.

## Methods

A retrospective analysis of consecutive patients who underwent hepatopancreatic surgery for cancer for a period of 12 months at La Maddalena Cancer Center was performed. This is a comprehensive cancer center with a high volume cancer surgery unit acting as a tertiary center for the entire region. Local ethical committee approval was obtained (Palermo 1, n.5/2023).

### Anesthesiological process

All procedures are routinely used by anesthetic team to provide homogeneous and optimal recovery after surgery allowing a safe discharge to surgical ward where they are continuously monitored for 24–48 h.

All patients were premedicated with fentanyl 50 µg, diazepam 5 mg, and dehydrobenzoperidol 2.5 mg and started gelatin infusion before performing epidural analgesia at T8-T10 to provide plasma expansion and prevent hypotension. After a test dose, 10 ml of lidocaine 2% was injected and a continuous epidural infusion of levobupivacaine 0.125% at 8–10 ml/h was started, preceded by a bolus of epidural morphine 2.5–3 mg. Anesthesia was induced by propofol, fentanyl, and rocuronium, and then maintained with desflurane, fentanyl, and rocuronium. A combination of neostigmine-atropine or sugammadex was used for the reversal of muscular relaxation. If epidural analgesia was contraindicated or not accepted by patients, intravenous postoperative analgesia with methadone was offered, after dose titration up to a successful dose in the recovery room [6]. Subsequent doses were administered every 8 h postoperatively for 48 h and then tapered according to the clinical needs. Patients were preferentially extubated, when possible, in the operating room, and then admitted to the recovery room (RR) for complete monitoring and appropriate warming with blankets. The occurrence of possible complications or symptoms in the recovery room (pain, nausea, shivering, and so on) was treated consequentially. When patients achieved an adequate modified Aldrete score [7], patients were discharged to the ward where they were monitored for 24–48 h. Otherwise, they were maintained under strict control in an intensive postoperative care unit. Postoperative pain was assessed regularly by means of a numerical scale 0–10, according to local nursing policy. Epidural catheters were removed 48 h after operation and intravenous/oral opioids were offered when necessary according to the needs.

### Data retrieved

Epidemiologic data were recorded. Duration of anesthesia, surgery, and RR staying were recorded. The need of invasive ventilation and its mean duration, the use of non-invasive ventilation (NIV) and its duration in the recovery room or eventually in the surgery unit were also recorded. The use of vasoactive drugs, including dobutamine and noradrenaline, red cell and plasma transfusions, global hospital stay, morbidity, and mortality were also recorded. Complications were graded according to the Dindo-Clavien classification [8].

### Statistical analysis

Statistical analysis of quantitative and qualitative data, including descriptive statistics, was performed for all items. Continuous data are expressed as mean ± SD, unless otherwise specified. Differences between groups were assessed by the chi-square test or Fisher exact test, as needed for categorical variables. The univariate analysis of variance (ANOVA) was performed for parametric variables. Data were analyzed by the Epi Info software (version 6.0, Centers for Disease Control and Prevention, Atlanta, GA, USA) and SPSS software (version 21.0, SPSS Inc, Chicago, IL, USA). All *P* values were two-sided and *P* values less than 0.05 were considered statistically significant.

## Results

A total of 163 patients were examined. Fifty-six patients underwent surgery for pancreatic cancer, and 107 patients underwent liver surgery for primary tumor or metastases (Table 1). The mean age of patients was 68.2 years (SD 12.4), and 77 patients (42.7%) were males. The median ASA physical status was 3, with 132 patients belonging to this category. The mean duration of anesthesia was 322 min (SD 320), and the mean duration of surgery was 296 min (SD 133). One-hundred-forty-seven anesthetics (90%) were blended anesthesia (general and epidural analgesia). The remaining interventions were performed under general anesthesia only. Extubation was performed in the operating room in 125 patients (76.7%). Post-operatory invasive ventilation was maintained in RR in fifteen patients for a mean duration of 72.7 min (SD 148.2), with one patient admitted to intensive care for 15 h. NIV was performed in three patients for a mean duration of 73.3 min (SD 15.3). The mean RR staying was 79 min (SD 80). In Table 1, data are presented according to the type of surgery. Data regarding the use of inotropic drugs in the postoperative period were available for 141 patients. Eighty-four of them (59%) received dobutamine (*n* = 50), noradrenaline (*n* = 19), or both dobutamine and adrenaline (*n* = 15) along the perioperative period for a mean period of 520 min (SD 689). Red cells transfusions were performed in 7 patients (mean 2.3 units, SD 1.1 for patient transfused). Plasma units were transfused in 2 patients (mean 7.5 units, SD 3.5 for patient). The mean global hospital stay was 10.3 days (SD 7.5), with a mean of 8.1 days (SD 5.7) of postoperative period. No complications were found in 144 patients. Principal postoperative complications were as follows: biliary leak requiring endoscopic intervention (1), biliary fistula (1), pancreatic-biliary fistula (1), ischemic cerebral attack (1), paralytic ileum (1), laparotomic dehiscence (1), ileostomy evisceration (1), wound infection (1), pneumothorax (1), lymphocele (1), liver abscess (1), peripancreatic blood collection (1) (see Dildo-Clavien classification in Table 1).

**Table 1:** *Pearson’s chi-square test or Fisher exact test, as needed; §Univariate analysis of variance (ANOVA)

	**Pancreas (56 pts)**	**Liver (107 pts)**	***P***
Age	67.9 (14.1)	68.4 (11.5)	0.798§
Gender (M/F)	24/32	53/54	0.418*
Duration of anesthetics (minutes)	416 (116)	272 (108)	<0.0005
Duration surgery (minutes)	386 (131)	250 (109)	<0.0005
Recovery room staying (minutes)	98 (131)	70 (24)	0.045
Invasive ventilation (n°)	6 pts	9 pts	0.776*
Mean duration (minutes)	141.7 (227.3)	26.7 (14.1)	0.147§
NIV (n°)	1 pt	2 pts	1.0*
Mean duration (minutes)	(60 min)	(80 min)	
Vasoactive drugs (n°)	33 pts	51 pts	0.189*
Mean duration (minutes)	1175 (732)	670 (609)	0.001§
Red cell transfusion (*n* pts, mean number)	4 pts	3 pts	0.233*
Plasma units (n° pts, n° unit)	1 pt	1 pt	1.0*
Hospital stay (days (SD))	15 (9.5)	7.9 (4.6)	<0.0005§
Postoperative stay (days (SD))	10.6 (8.5)	6.8 (2.7)	<0.0005§
Dindo-Clavien			
0	46	101	0.023*
I	4	3	0.233*
II	2	0	0.116*
III	0	3	0.551*
V	4	0	0.012*
Mortality	4	1	0.002*

## Discussion

In the last decades, improvements in surgical techniques and perioperative management have dramatically improved the outcome of major abdominal surgery for cancer [9]. In this study, fast-track anesthesia allowed to transfer most patients to the surgical ward, after staying in RR until adequate stabilization was achieved. While the use of colloids remains controversial, the use of gelatin was consolidated for years at our institution, preventing hypotension after an epidural lidocaine bolus at start of induction, while providing a moderate hemodilution to prevent the use of red cell transfusion that was fairly low. This approach resulted in a good postoperative outcome, avoiding admission to intensive care despite the duration of major abdominal cancer surgery. This result allowed a better availability of intensive care beds and limitation of costs, maintaining an acceptable outcome for patients. In fact, only one patient required an admission to intensive care for just 1 day, despite almost patients were ASA 3.

In recent years, several forms of enhanced recovery protocol for major abdominal cancer surgery have been proposed. In comparison with data reported about 10 years ago in a Scandinavian country [10], or large series of hepatic resections [11], this approach allowed a median postoperative length stay of 6 days [12], although these authors also included laparoscopic procedures. In another study, the median postoperative length stay was 5 days, although patients with ASA status of 3 were managed in an intensive care on the first postoperative night [2]. The median stay following pancreatic resection has been reported to be higher, about 16 days [13]. Optimal pain management is one of the most important factor to ameliorate postoperative recovery after liver or pancreatic surgery [14], possibly decreasing the length of stay after surgery. Perioperative mortality following pancreatic surgery has been reported to have an incidence of 1–4%, while morbidity still remains high [15]. In this study, mortality rate was 7.1% and 0.9% for pancreas and liver surgery, respectively. The quality standard for pancreatic surgery is mortality <10% and morbidity <50%, but centers with a high volume of patients exhibit mortality <5% and even around 10% [16–18]. In Italy, mortality rate is related to high and medium-low volumes, being 5% and 10%, respectively [19, 20]. In these patients, the mortality rate was higher than patients undergoing hepatic surgery. This data was expected. Mortality and complications of pancreatic surgery occur more commonly in elderly patients [21]. Again, almost all patients were ASA 3, and death was not attributable to the fast-track protocol. Overall in-hospital mortality was 9.5%, with leak of the pancreato-jejunal anastomosis being the most frequent complication (16%) [22], although some other experiences reported in a high volume center, mortality rate was 2.1% [23].

Limitations of the study are represented by its retrospective nature. However, the quality of data collection was sufficient to gather useful information about cancer patients undergoing pancreatic and liver surgery.

## Conclusion

A program of fast-track anesthesia with a short stay in recovery room allowed to achieve a good outcome, limiting the costs of intensive care admission. Complications were not related to discharge to the unit.

## Data Availability

Data are available on request.

## References

[1] Kehlet H, Dahl JB (2003). Anaesthesia, surgery and challenges in postoperative recovery. Lancet.

[2] Schultz NA, Larsen PN, Klarskov B, Plum LM, Frederiksen HJ, Christensen BM, Kehelet H, Hillingson JG (2013). Evaluation of a fast-track programme for patients undergoing liver resection. Br J Surg.

[3] Jarnagin WR, Gonen M, Fong Y (2002). Improvement in perioperative outcome after hepatic resection. Ann Surg.

[4] Melloul R, Larssen K, Roulin D, Grass F, Perinel J, Adham M, Wellge EB, Kunzler F, Besselink MG, Asbun H, Scott MJ, Dejong CH, Vrochides D, Aloia T, Izbicli  J, Demartines N (2020). Guidelines for perioperative care for pancreatoduodenectomy enhanced recovery after surgery (ERAS) recommendations 2019. World J Surg.

[5] Lillemoe HA, Aloia TAM (2018). Enhanced recovery after surgery: Surg Clin North Am Hepatobiliary..

[6] Mercadante S, David F, Villari P, Spedale VM, Casuccio A (2020). Methadone versus morphine for postoperative pain in patients undergoing surgery for gynecological cancer: a randomized controlled clinical trial. J Clin Anesth..

[7] Aldrete JA (1995). The post-anesthesia recovery score revisited. J Clin Anesth..

[8] Dindo D, Demartines N, Clavien PA (2004). Classification of surgical complications. A new proposal with evaluation in a cohort of 6336 patients and results of a survey. Ann Surg.

[9] Lentschener C, Ozier Y (2002). Anaesthesia for elective liver resection: some points should be revisited. Eur J Anaesthesiol.

[10] Jensen LS, Mortensen FV, Iversen MG, Jorgensen A, Kirkegaard P, Kehlet H (2009). Liver surgery in Denmark 2002–2007. J Ugeskr Laeger.

[11] Kamiyama T, Nakanishi K, Yokoo H, Kamachi H, Tahara M, Kakisaka T, Tsuruga Y, Todo S, Taketomi A (2012). Analysis of the risk factors for early death due to disease recurrence or progression within 1 year after hepatectomy in patients with hepatocellular carcinoma. World J Surg Oncol.

[12] Savikko J, Ilmakunnas M, Makisalo H, Nordin A, Isoniemi H (2015). Enhanced recovery protocol after liver resection. Br J Surg.

[13] Cunningham JD, O’ Donnell N, Starker P.  (2009). Surgical outcomes following pancreatic resection at a low-volume community hospital: do all patients need to be sent to a regional cancer center?. Am J Surg.

[14] Ntinam A, Kardassis D, Kostantinopoulos I, Kottos P, Manias A, Kyritsi M, Zilianiak D, Vrochides D (2013). Duration of thoratic epidural catheter in a fast-track receovery protocol may decrease the length of stay after major hepatectomy: a case control study. Int J Surg.

[15] Spanknebel K, Conion KC (2001). Advances in the surgical management of pancreatic cancer. Cancer J.

[16] Grutzmann R, Ruckert F, Hippe-Davies N, Distler M, Saeger HD (2012). Evaluation of the International Study Group of Pancreatic Surgery definition of post-pancreatectomy hemorrhage in high-volume center. Surgery.

[17] Simons JP, Shah SA, Chau S, Whalen GF, Tseng JF (2009). National complications rates after pancreatectomy: beyond mere mortality. J Gastroint Surg..

[18] Sohn TA, Yeo CJ, Cameron JL, Koniaris L, Kaushal S, Abrams RA, Sauter PK, Coleman J, Hruban RH, Lillemoe KD (2000). Resected adenocarcinoma of the pancreas - 616 patients: results, outcomes, and prognostic indicators. J Gastrointest Surg..

[19] Balzano G, Capretti G, Callea G, vantù E, Carle F, Pezzilli R.  (2016). Overuse of surgery in patients with pancreatic cancer. A nationwide analysis in Italy. HPB (Oxford).

[20] Balzano G, Guarneri G, Pecorelli N, Reni M, Capurso G, Falconi M (2021). A four-step method to centralize pancreatic surgery, accounting for volume, performance and access to care. HPB (Oxford).

[21] Riall TS (2009). What is the effect of age on pancreatic resection?. Adv Surg.

[22] Metreveli RE, Sahm K, Abdel-Misih R, Petrelli NJ (2007). Major pancreatic resections for suspected cancer in a community-based teaching hospital: lessons learned. J Surg Oncol.

[23] Fontes PR, Waechter FL, Nectoux M (2014). Low moratlity rate in 97 consecutive pancreaticoduodenectomies: the experience group. Arq Gastroenterol.

